# Intravertebral cleft in pathological vertebral fracture resulting from spinal tuberculosis: a case report and literature review

**DOI:** 10.1186/s12891-020-03642-2

**Published:** 2020-09-18

**Authors:** Liang Dong, Chunke Dong, Yuting Zhu, Hongyu Wei

**Affiliations:** 1 Department of Spine Surgery, Honghui Hospital, Xi’an Jiaotong University, No 555, YouYi East road, Xi’an, 710054 China; 2 Beijing University of Chinese Medicine, 11 North Third Ring Road East, Chaoyang District, Beijing, 100029 China; 3 Beijing Tongzhou Integrative Medicine Hospital, 89 Chezhan Road, Tongzhou District, Beijing, 101100 China; 4 Department of Orthopaedic Surgery, China-Japan Friendship Hospital, 2 Yinghuadong Road, Chaoyang District, Beijing, 100029 China

**Keywords:** Intravertebral cleft, Vertebral fracture, Spinal tuberculosis, Case report

## Abstract

**Background:**

Among common findings in osteoporotic vertebral compression fractures (OVCFs), the intravertebral cleft (IVC) is usually considered a benign lesion. The current study was aimed to present a rare case of vertebral fracture caused by IVC-related spinal tuberculosis.

**Case presentation:**

A 73-year-old female complained of back pain and weakness in lower limbs for 2 weeks. 3 months ago, after a minor trauma, she got back pain without weakness in lower limbs. Initially, she was diagnosed with a L1 compression fracture and accepted conservative treatment. After an asymptomatic period, she complained progressive pain at the fracture position with weakness of both lower limbs and was referred to our hospital with suspicion of Kümmell’s disease. The patient underwent posterior debridement and internal fixation for decompression and stabilization of the spine. Pathological examinations revealed the patient with spinal tuberculosis.

**Conclusions:**

Although IVC is common in patients with OCVFs, there are some cases believed to be found in patients with spinal tuberculosis or infection. Further test, like CT-guided puncture biopsy, may be required before decisive treatment when an IVC is observed.

## Introduction

The intravertebral cleft (IVC), which was first described by Maldague in 1978, has long been considered the result of local bone ischemia associated with nonunion vertebral collapse [1]. Patients with IVC often present with a transverse, linear or semilunar radiolucent shadow, indicating the collection of air inside the vertebral body [1, 2]. However, several studies have also observed fluid accumulation within non-healing intervertebral clefts in patients with benign OVCFs, which depends on the position of the patient secondary to the extension momentum in the supine position [3, 4].

Although several studies have found that IVC can be found in pathologically fractured vertebrae caused by infections, multiple myeloma and malignant tumors [5–8], IVC is highly suggestive of a benign lesion due to rare reports. We retrospectively reviewed the imaging data containing X-rays, CT, and MRI of 149 consecutive patients with IVC. Among them, 46 patients underwent a spinal reconstructive surgery and intraoperative biopsy. To the best of our knowledge, few studies have reported detailed pathological results and treatment for vertebral fracture caused by IVC-related spinal tuberculosis. The aim of presenting the rare case is to raise clinicians’ awareness of the possibility of IVC in pathological vertebral fracture attributable to spinal tuberculosis or infection.

## Case presentation

### Medical history

**Fig. 1:**
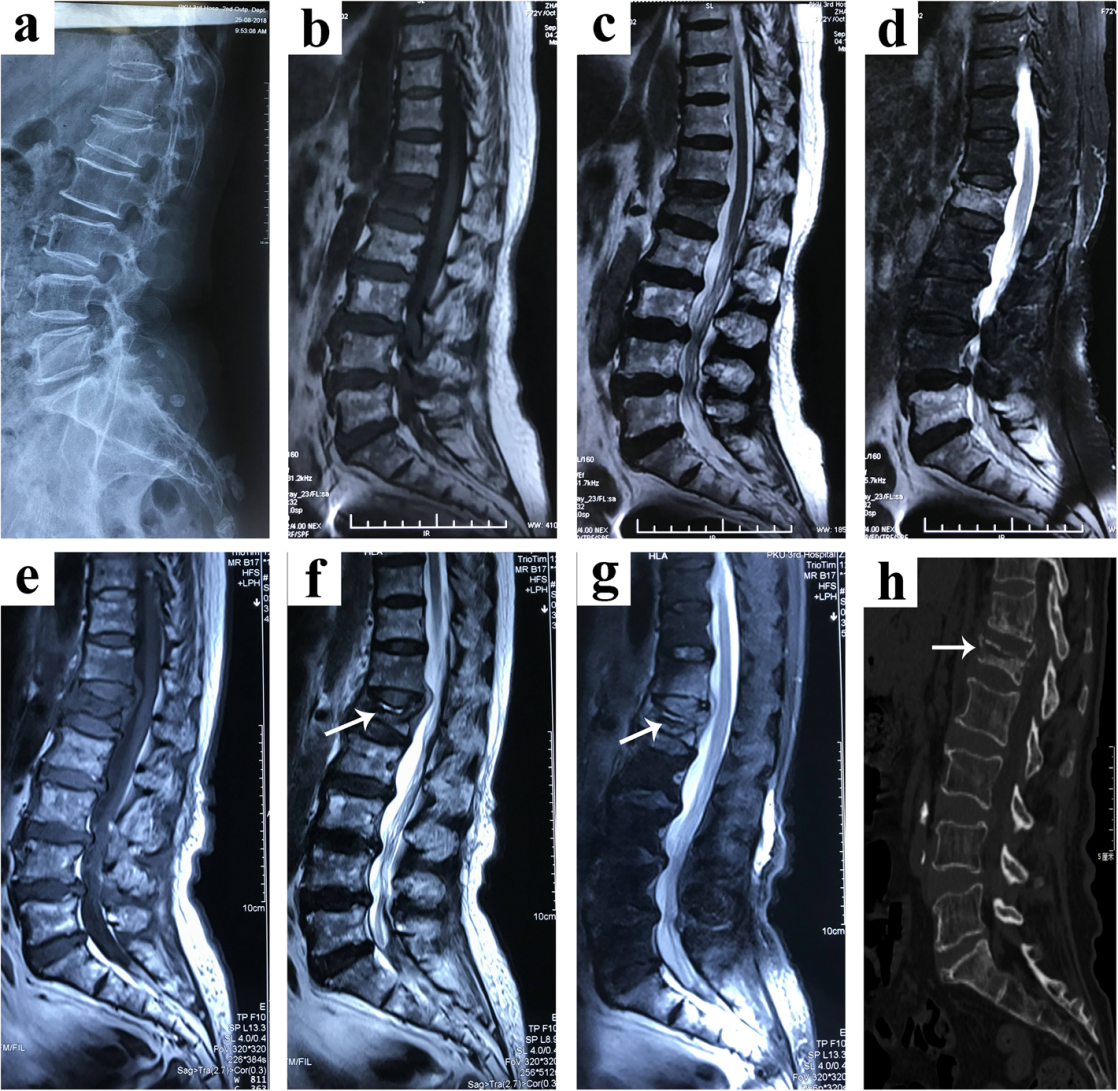
Preoperative X-ray, CT and MRI of the patient. **a** lateral radiographs show a vertebral collapse of L1 three months before surgery. **b-d** Sagittal MR images of lumbar spine display L1vertebral collapse without IVC three months before surgery. **e-f** Preoperative sagittal MR images show a fluid-containing IVC with high-signal intensity on T2-weighted images and STIR MR sequences. **h** Preoperative sagittal reconstruction CT scan shows a linear radiolucent shadow that is located adjacent to the upper endplate of L1collapsed vertebral body

A 73-year-old female complained of back pain and weakness in lower limbs for 2 weeks. 3 months ago, after a minor trauma, she got back pain without weakness in lower limbs. Radiography including lateral radiographs and MRI (Fig. 1a-d) performed at a local hospital. Initially, she was diagnosed with a L1 compression fracture and accepted conservative treatment. After an asymptomatic period, she complained progressive pain at the fracture position with weakness of both lower limbs and was referred to our hospital with suspicion of Kümmell’s disease. The back pain evaluated by visual analog scale (VAS) scale was 9. According to American Spinal Injury Association (ASIA) grading criteria, the neurological function was rated as ASIA C. Sagittal MR images showed a fluid-containing IVC with high-signal intensity on T2-weighted images and STIR MR sequences at L1 (Fig. 1e-g) and sagittal reconstruction CT scan (Fig. 1h) showed a linear radiolucent IVC, accompanying spinal cord compression. The biochemical workup revealed no abnormal indications of infection (including C-reactive protein (CRP) levels, erythrocyte sedimentation rate (ESR) and T-SPOT). Furthermore, the patient denied the history of cancer or tuberculosis and she also denied hypothermia, night sweats and weakness.

### Surgical treatment

**Fig. 2:**
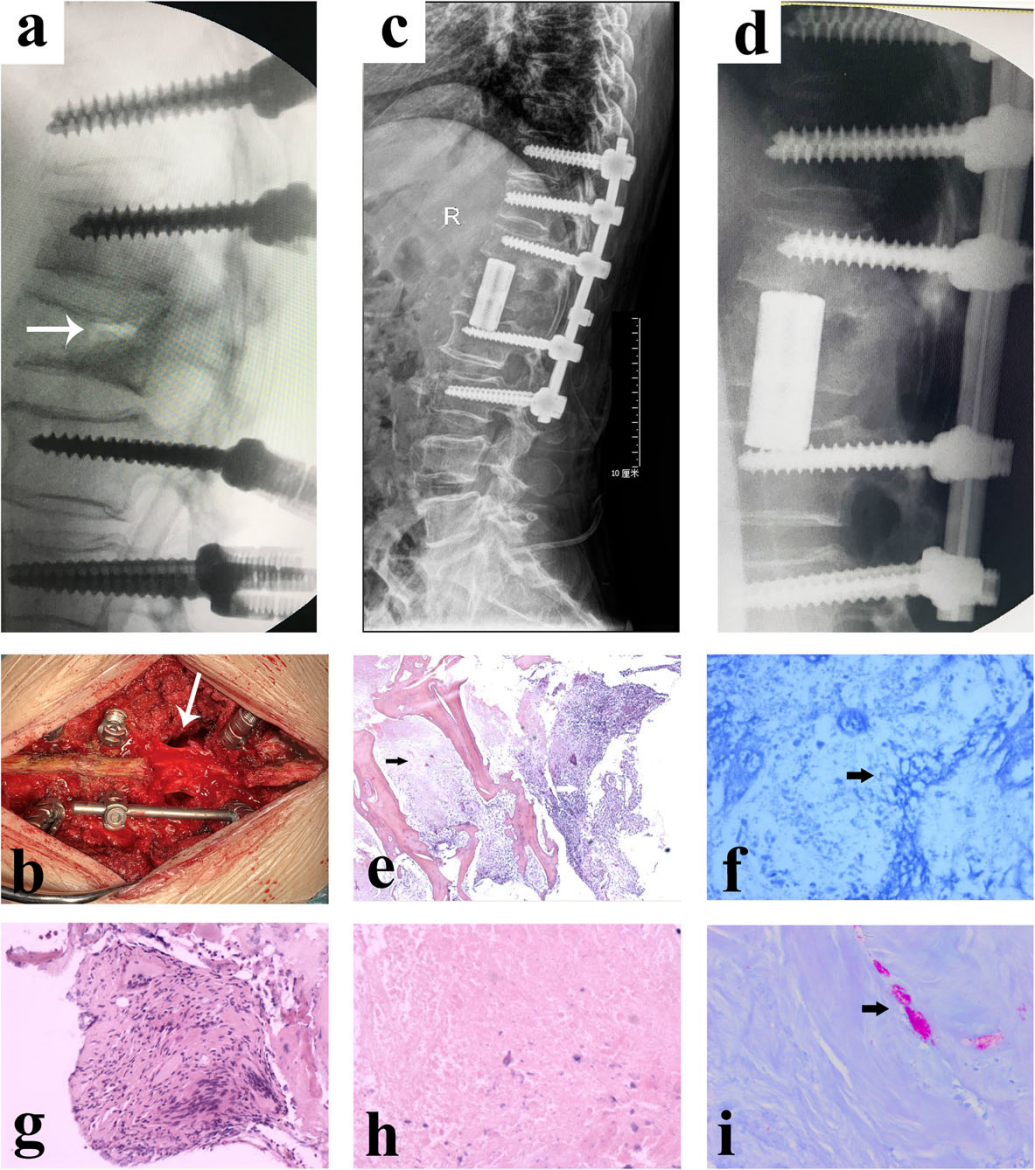
Intra- and post-operative radiographs (**a**-**d**). a Intraoperative lateral radiographs showed a linear IVC in L1(*white arrow*). **b-c** Posterior artificial vertebral body implantation with osteotomy and debridement at L1(*white arrow*). **d** Postoperative lateral radiograph shows good positioning of artificial vertebral body and pedicle screws. Pathological results (**e-i**). **e** Necrotic tissue (*black arrow*) and hematopoietic tissue (*white arrow*) (H & E stain, original magnification × 4). **f** Acid-fast bacillus (+) (*black arrow*) (acid-fast staining, original magnification × 40). **i** Acid-fast bacillus (+) (*black arrow*) (acid-fast staining, original magnification × 20). **g** epithelioid granuloma (H & E stain, original magnification × 20). **h** Caseous necrosis (H & E stain, original magnification × 40)

Before surgery, we obtained the informed consent of the patient and her family to perform the operation. The patients were placed in a prone position under general anesthesia with somatosensory-evoked potentials and motor-evoked potentials for spinal cord monitoring. After the lesion was positioned with the C-arm, a standard posterior midline approach with subperiosteal stripping was used to expose the spinous processes, lamina, and facet joints. Considering the presence of osteoporosis in elder female patient, we performed a long segment pedicle screw fixation (Cox Spinal Screw-Rod System, Fule Science & Technology, Beijing, China) from T10-L3 to avoid implant failure (Fig. 2a). Then, a complete laminectomy-facetectomy was performed to decompress and fully visualize the dural sac. A temporary stabilizing rod was fixed on one side of the pedicle screws. On the contralateral side, the facet joints of the diseased vertebra were removed to reveal the pedicle. Then, the pedicle and vertebral body, including the superior and inferior disk, were piecemeal removed by rongeurs, osteotomes, or curettes. To our surprise, caseous necrosis and inflammatory granulation could be seen in the surgically resected lesions. The specimens were sent for pathologic examination. After osteotomy and debridement in anterior column at L1, we used a ‘off-the-shelf’ (OTS) three-dimensional (3D) printed artificial vertebral body (Fig. 2b-c, Beijing AK Medical Co., Ltd.) instead of various materials, such as bone grafts, mesh cages, or expandable titanium cages, to reconstruct sagittal alignment [9]. Based on preoperative 3D reconstruction of CT and MRI images, the artificial prosthesis was designed in conformity with the expected defects that may occur after the affected vertebral body resection.

### Pathologic results

Pathological examinations reported caseous necrosis tissue, epithelioid granuloma existed with the hyperplasia tissue and the acid-fast bacillus was also found (Fig. 2e-i).

### Surgical outcomes

After surgery, the patient was treated with quadruple anti-tuberculous chemotherapy and hepatoprotective drug for 12 months and required to wear a brace for at least 3 months. Two weeks after surgery, the patient could walk and discharge from the hospital. Three months after surgery, the VAS scores decreased from preoperative 9 to 1 and the neurological function recovered to ASIA E. No internal fixation failure and recurrence of tuberculosis occur at last follow-up (Fig. 2d).

## Discussion

IVC is commonly considered to be a sign of avascular necrosis in patients with osteoporotic vertebral compression fractures, and is a widely reported radiological sign associated with Kümmell’s disease, a not rare reported clinical phenomena as a result of delayed posttraumatic vertebral collapse (incidence 10–48%) [10–12]. Prior reports have also shown that IVC is an indication of benign vertebral fractures due to damage of the microtrabecular structure of the respective vertebrae [3]. Although the pathogenesis of IVC remains unclear, the image characteristics of IVC have been generally accepted by surgeons: (1) The IVC appears on radiographs as a transverse, linear or semilunar radiolucent shadow that is located centrally within or adjacent to the endplate of a collapsed vertebral body [1]. (2) On CT scans, the sign may appear more heterogeneous and irregular than on radiographs and the diagnostic sensitivity of IVC on CT scans is higher than radiographs [11]. (3) On MR images, an air-containing IVC is generally seen as low signal intensity with T1-weighted, T2-weighted and/or short-tau-inversion-recovery (STIR) sequences. However, a fluid-containing IVC shows high-signal intensity on T2-weighted images and/or STIR MR sequences. Whether air or fluid was presented on MR images mainly depended on the time of examination and the position of patients [13].

In recent years, a few cases of IVC have been identified in patients with multiple myeloma and cancer metastasis, which were similar to those observed in OVCFs, making it difficult to differentiate between these two types of IVC [7, 8]. In terms of IVC resulting from infection, several case reports suggested that the gas observed in vacuum phenomena may be produced directly by gas-forming organisms which was different from the pathogenesis in OVCFs [5, 7]. The distribution of IVC in tuberculous spondylitis is uneven, bubble-like, even extends to the paravertebral soft tissue [7]. The differences IVC observed between patients with OVCFs and infection or metastasis may be due to several factors. First, vertebral collapse during infection or metastasis may actually represent bony destruction or erosion, but not a real fracture, which usually has two or more fracture fragments. Therefore, the opening–closing mechanism may not occur in a collapsed vertebral body, thereby preventing the formation of negative pressure. Second, tissue inflammation during an active infection may promote fluid accumulation and tissue swelling, resulting in positive pressure at the lesion site [7]. However, in our case, IVC appears as a linear shadow on the X-ray image, near the upper endplate of the affected vertebral body. Unlike normal imaging characteristics about spinal infections or tuberculosis with endplate destruction or disc space narrow in plain radiographs, necrotic and reactive bone form in CT scans and adjacent vertebral bodies signal changing in MRI [7], the imaging features of IVC in this case are similar to those in OVCFs. Initially, this patient was diagnosed Kümmell’s disease, a clinical syndrome characterized by minor trauma with a symptom-free period from months to years, which was consistent with our patient’s condition. And the indicators of infection such as CRP, ESR and T-SPOT were negative.

Most patients with IVC occur as a benign lesion in OVCFs and do not respond well to further conservative treatment. Vertebral augmentation, including percutaneous vertebroplasty (PVP) and percutaneous balloon kyphoplasty (PKP) have been demonstrated to be minimally invasive and effective in treating OVCFs with IVC [12–14]. Although the similarity may cause the initial misdiagnosis or delayed diagnosis of IVC in multiple myeloma or cancer metastasis, reports have shown that the pain caused by pathological vertebral collapse in multiple myeloma or cancer metastasis can still be managed via vertebroplasty [15, 16]. However, active infection has been considered to be an absolute contraindication for vertebroplasty [17, 18].

Initially, the patient was misdiagnosed as Kümmell’s disease with neurological deficits. Owing to the progressive kyphosis and intravertebral instability at the cleft site, patients with advanced-stage Kümmell’s disease are more susceptible to neurological deficits [19], which is a relative contraindication for cement usage [20]. In recent years, short-segment pedicle screw fixation with polymethylmethacrylate (PMMA) augmentation has been employed for Kümmell’s disease complicated by neurological deficits [21–24], however, some scholars find this procedure may not be supportive enough for the long-term stabilization effect [19]. Therefore, considering serious comorbidities and severe osteoporosis in elderly patients, one-stage posterior osteotomy and fixation is more suitable for treating Kümmell’s disease with neurological deficits compared with anterior or anterior and posterior approaches for a long-term effect. Thus, we performed a one-stage posterior vertebral column resection and internal fixation for spinal cord decompression and reconstruction of spinal stability [19, 20, 25].

According to reports, the incidence of implant-related complications is from 14.3 to 21.6% in anterior reconstruction surgery for Kümmell’s disease [26] and cage subsidence is an important risk factor related to the instrumentation failure [27]. In the past few years, 3D printed artificial vertebral body with good implant fit and less subsidence has gained traction in spine surgery [28–30] and the excellent result may attribute to the following aspects: (1) a larger diameter endcap of 3D prosthesis allows for an expansion of the bone-implant interface, which distributes point-loading and loads the periphery of the endplates where there is thicker cortical bone, and eventually reduces the risk of subsidence [29, 31]; (2) with a Young‘s modulus more similar to native human bone (0.5–20 GPa), may reduce subsidence and ‘stress shadowing effect’ compared with traditional implants [32]; (3) the porosity of 3D vertebral body made by Ti6-Al4-V titanium alloy can enhance the delivery of osteoinductive factors as well as facilitate osteoconduction, thus potentially improving bony ingrowth [29].

A patient-specific 3D prosthesis has been explored to fit the unique spinal pathoanatomy of complex congenital, traumatic and neoplastic pathologies [33], however, requires extensive design and manufacturing processes prior to production, which spends a lot of money and time [29]. In our study, the lesion segments were located in the thoracolumbar segment with less adjacent segment degeneration, so there was no need to use a custom prosthesis. The OTS produced by Beijing AK Medical Co., Ltd. and used in our study, could provide sufficient angle and height to fit with adjacent vertebrae based on the preoperative CT measurement. Thus, we employed an OTS 3D printed prosthesis for anterior column reconstruction. Although the patient may be diagnosed with tuberculosis intraoperatively, we still used the original surgical plan for debridement. Moreover, it is appropriate to use 3D printing vertebrae in this patient for titanium alloy could furthermore minimize bacterial adhesion and biofilm formation [34, 35]. Although no cage subsidence, broke, and migration occurred 12 months after surgery, further studies will be needed to reveal long-term reconstruction outcomes of the 3D printed prosthesis.

In conclusion, although our case report is an incidental finding and IVC is common in OCVFs, there are some IVC believed to be found in spinal infection or malignancy. Thus, further tests, such as MRI or CT-guided needle biopsy, may be necessary before planning further treatment when an IVC is observed.

## Data Availability

All data used or analyzed during this study are included in this published article.

## Abbreviations

IVC: Intravertebral cleft
OVCFs: Osteoporotic vertebral compression fractures
VAS: Visual analog scale
ASIA: American Spinal Injury Association
CRP: C-reactive protein
ESR: Erythrocyte sedimentation rate
PVP: Percutaneous vertebroplasty
PKP: Percutaneous balloon kyphoplasty
OTS: Off-the-shelf
